# Dependência de Pacing a Longo-Prazo e Preditores de Implante de Pacemaker após Implante Percutâneo de Prótese Valvular Aórtica – 1 Ano de Seguimento

**DOI:** 10.36660/abc.20210613

**Published:** 2022-07-13

**Authors:** Ricardo Alves Pinto, Tânia Proença, Miguel Martins Carvalho, Gonçalo Pestana, Ana Lebreiro, Luis Adão, Filipe Macedo

**Affiliations:** 1 Departamento de Cardiologia Centro Hospitalar Universitário de São João Porto Portugal Departamento de Cardiologia, Centro Hospitalar Universitário de São João, E.P.E., Porto – Portugal; 2 Faculdade de Medicina Universidade do Porto Porto Portugal Faculdade de Medicina da Universidade do Porto, Porto – Portugal

**Keywords:** Estenose Aórtica, Bloqueio Atrioventricular, Substituição Valva Aórtica Transcateter, Implante Marcapasso, Implante Prótese Valvar Aórtica, Distúrbios de Condução Cardíaca

## Abstract

**Fundamento:**

Os distúrbios de condução (DC) são a complicação mais frequente após a substituição da válvula aórtica transcateter (TAVR) e ainda não há consenso sobre seu tratamento.

**Objetivo:**

Avaliar novos DC e implante de marca-passo definitivo (MPD) após a TAVR e avaliar a porcentagem de estimulação ventricular (EV) até 1 ano de acompanhamento.

**Métodos:**

Pacientes submetidos a TAVR de outubro de 2014 a novembro de 2019 foram cadastrados; pacientes com MPD anterior foram excluídos. Dados clínicos, do procedimento, do ECG e do MPD foram coletados até 1 ano após o implante. O nível de significância adotado para a análise estatística foi 0,05%.

**Resultados:**

Um total de 340 indivíduos foram submetidos a TAVR. O DC mais comum foi bloqueio de ramo esquerdo novo (BRE; 32,2%), sendo que 56% destes foram resolvidos após 6 meses. O bloqueio do ramo direito (BRD) foi o maior fator de risco para bloqueio atrioventricular avançado (BAV) [RC=8,46; p<0,001] e implante de MPD [RC=5,18; p<0,001], seguido de BAV de baixo grau prévio [RC=2,25; p=0,016 para implante de MPD]. Em relação às características do procedimento, válvulas de gerações mais recentes e procedimentos de válvula-em-válvula foram associados a menos DC. No total, 18,5% dos pacientes tiveram MPD implantado após a TAVR. Na primeira avaliação do MPD, pacientes com BAV avançado tinham uma porcentagem mediana de EV de 80%, e, após um ano, de 83%. Em relação aos pacientes com BRE e BAV de baixo grau, a EV mediana foi mais baixa (6% na primeira avaliação, p=0,036; 2% após um ano, p = 0,065).

**Conclusão:**

O BRE foi o DC mais frequente após a TAVR, com mais da metade dos casos se resolvendo nos primeiros 6 meses. O BRD foi o principal fator de risco para BAV avançado e implante de MPD. O BAV avançado foi associado a uma porcentagem mais alta de EV no acompanhamento de 1 ano.

## Introdução

A substituição da válvula aórtica transcateter (TAVR) é um procedimento bem estabelecido para o tratamento de pacientes com estenose aórtica grave sintomática com risco cirúrgico aumentado ou proibitivo. O aumento da experiência levou ao crescimento da consideração da TAVR como uma opção para as pessoas com risco mais baixo.^1 - 3^ A adoção generalizada da TAVR foi seguida de uma redução na maioria das complicações periprocedurais, exceto pelos novos distúrbios de condução e consequente necessidade de implante de MPD.^1 , 4 , 5^ Novos BRE, com uma incidência de cerca de 25% (4 a 65%), são os distúrbios de ritmo mais frequentemente documentados após a TAVR e provavelmente os mais desafiadores.^1^ Embora seja frequentemente autolimitado, uma porcentagem significativa desses pacientes desenvolve BAV avançado ou bloqueio cardíaco total, as complicações de condução pós-TAVR mais graves.^1 , 2 , 4 , 6 , 7^

Ainda há questões importantes sobre o tratamento dos distúrbios de condução após a TAVR, levando a abordagens diferentes entre os vários centros. Geralmente, os pacientes permanecem monitorados como telemetria e eletrocardiogramas (ECG) diários após o procedimento, às vezes com marca-passo reserva temporário, aumentando o período de internação e os custos do procedimento.^4 , 7 , 8^ Há dados limitados sobre os fatores de risco para o desenvolvimento de BAV avançado e a necessidade de se manter um marca-passo temporário, o que também se traduz na variação dos índices de implante de MPD pós-TAVR.^1 , 7^

O objetivo do presente estudo foi descrever novos distúrbios de condução e implante de MPD em pacientes submetidos a TAVR com uma prótese valvar balão-expansível ou autoexpansível. Também foi avaliada a porcentagem de EV em pacientes que foram submetidos a implante de MPD em até um ano de acompanhamento.

## Métodos

### População do estudo

O presente estudo incluiu uma amostra de pacientes consecutivos submetidos a TAVR no Centro Hospitalar Universitário de São João, E.P.E., um centro terciário no Porto, Portugal, de outubro de 2014 a novembro de 2019 (n = 371). Pacientes que tinham MPD antes do implante da válvula foram excluídos (n = 31). Os demais 340 pacientes foram analisados retrospectivamente. Dados clínicos, eletrocardiográficos, ecocardiográficos e do procedimento foram coletados na apresentação e até 1 ano após o implante, incluindo a interrogação sistemática de MPD implantados. Este estudo foi aprovado pelo comitê de ética institucional.

### Definições, dados e coleta de ECG

Os desfechos clínicos e a definição de distúrbios de condução estavam de acordo com o Consenso do Valve Academic Research Consortium (Consórcio de pesquisa acadêmica valvar) (VARC)-2 e o consenso do JACC Scientific Expert Panel (Painel científico de especialistas do JACC), respectivamente.^1 , 9^ Os ECG foram obtidos sistematicamente na linha de base (geralmente no dia anterior à TAVR), imediatamente após o implante da válvula (na admissão na unidade de tratamento cardiológico) e pelo menos uma vez ao dia até a alta hospitalar. Todos os pacientes tiveram monitoramento eletrocardiográfico durante a internação hospitalar. A maioria dos ECG em nossa instituição foram registrados eletronicamente e foram avaliados e analisados por cardiologistas. Os dados clínicos, ecocardiográficos e do procedimento foram obtidos de registros digitais. O BAV de baixo grau foi definido como BAV Mobitz I de 1º ou 2º graus. O BAV avançado foi definido como BAV Mobitz II de 2º grau ou de 3º grau.

### Procedimento

Pacientes submetidos a TAVR com válvulas autoexpansíveis (Medtronic CoreValve, Medtronic CoreValve Evolut R, Medtronic CoreValve Evolut Pro, Boston Scientific Acurate Neo, Abbott Portico e Boston Scientific LOTUS) e balão-expansíveis (Edwards SAPIEN 3) foram incluídos. Todos os pacientes tiveram um cateter de estimulação transvenoso temporário colocado no ventrículo direito. Dependendo do novo surgimento de distúrbios de condução ou risco de distúrbio de ritmo pré-procedimento, e de acordo com o consenso do JACC Scientific Expert Pane,^1^ o marca-passo temporário foi retirado imediatamente no laboratório de cateterização ou posteriormente durante a internação hospitalar (geralmente 24 – 48h). Para fins deste estudo, a análise de válvulas de nova geração incluiu procedimentos com as válvulas SAPIEN 3, CoreValve Evolut Pro e Acurate Neo, enquanto as demais foram classificadas como válvulas de geração anterior.

### Indicação de marca-passo definitivo e acompanhamento

Os MPD foram implantados de acordo com as diretrizes ACC/AHA/HRS de 2018 para bradicardia e retardo de condução cardíaca e de acordo com o JACC Scientific Expert Panel.^1 , 10^ Todos os dispositivos foram analisados no 1º e no 7º dia após o implante. A condução intrínseca de VA foi sistematicamente questionada e foram aplicados algoritmos para minimizar a EV (modo de estimulação ventricular gerenciada ou modo de estimulação AAI com reserva de VVI na maioria dos pacientes). Para fins deste estudo, a primeira avaliação do MPD foi definida como primeira avaliação do dispositivo após a alta (tempo mediano 3 meses após o implante, FIQ 3 - 4 meses) e a avaliação de um ano foi definida como a segunda avaliação ambulatorial do dispositivo (tempo mediano de 12 meses após o implante, FIQ 10 - 12 meses). Como alguns pacientes foram acompanhados em outras instituições médicas, os dados do acompanhamento do MPD não estavam disponíveis para 30% e 43% dos pacientes para a primeira avaliação do MPD e para a avaliação de um ano, respectivamente.

### Análise estatística

Os dados foram expressos como mediana (faixa interquartil [FIQ]) para variáveis contínuas e porcentagens para variáveis categóricas. O teste de Kolmogorov-Smirnov de uma amostra foi realizado para avaliar a distribuição normal. Variáveis categóricas foram comparadas pelo teste Qui-quadrado, e razões de chance (RC) são apresentadas quando se considera relevante. As variáveis contínuas foram comparadas usando-se o teste U de Mann-Whitney. Diferenças com um p valor = <0,05 foram consideradas estatisticamente significativas. A análise estatística foi realizada no IBM SPSS Statistics versão 25.

## Resultados

### População do estudo

Um total de 340 pacientes submetidos a TAVR entre outubro de 2014 e novembro de 2019 foram incluídos nesta amostra, após a exclusão de 31 pacientes com MPD prévio.

As características de linha de base da população do estudo estão resumidas na tabela 1 e na tabela 2 . A idade mediana foi de 81 anos (FIQ 76 a 85 anos) e 57% dos pacientes eram mulheres.

**Tabela 1:** Linha de base

N	340
Idade, anos (FIQ)	81 (76 - 81)
Feminino (%)	193 (57)
Hipertensão (%)	294 (87)
Diabetes (%)	127 (37)
Dislipidemia (%)	244 (72)
Doença renal prévia (%)	185 (62)
em diálise (%)	10 (3)
Fibrilação atrial (%)	78 (23)
Função do VE preservada (%)	244 (73)
Válvula bicúspide (%)	8 (3)
Área da válvula aórtica (FIQ)	0,7 cm^2^ (0,6 – 0,9)
Gradiente de pressão transvalvar (FIQ)	46 mmHg (39,5 - 59)
Fração de ejeção VE (FIQ)	60 % (44 - 65)
Regurgitação aórtica grave (%)	15 (6)

*A tabela 1 apresenta as características de linha de base da população do estudo. Os valores foram apresentados como mediana (FIQ) ou número de casos (%). FIQ: faixa interquartil; anos - idade; VE: ventrículo esquerdo.*

**Tabela 2:** Características de ritmo pré-TAVR

Ritmo	
Ritmo sinusal	262 (77)
Fibrilação atrial	78 (23)
**Condução AV**	
Condução AV normal	207 (79)
BAV de 1º grau	53 (20)
BAV Mobitz I de 2º grau	2 (1)
**Condução IV**	
BRE	31 (9)
BRD	25 (7)
Bloqueio fascicular anterior esquerdo	24 (7)
Bloqueio bifascicular	23 (7)
Retardo de condução intraventricular não específico	33 (10)

*A tabela 2 resume o ritmo cardíaco, a condução atrioventricular (AV) e a condução intraventricular (IV) da população do estudo antes da TAVR. A condução AV foi considerada apenas no ritmo sinusal. Os valores foram apresentados como número de casos (%). BAV: bloqueio atrioventricular; BRE: bloqueio de ramo esquerdo; BRD: bloqueio de ramo direito.*

Na linha de base, 77% dos pacientes estavam em ritmo sinusal e 23% tinham FA. A maioria dos pacientes que estavam em ritmo sinusal tinha condução atrioventricular (AV) normal. Em relação à condução intraventricular (IV), 60% não tinham distúrbio de condução e o distúrbio mais frequente foi um retardo de condução intraventricular não específico (NICD; tabela 2 ).

A válvula autoexpansível CoreValve Evolut R foi a usada mais frequentemente (41% dos casos), seguida das válvulas CoreValve Evolut Pro e Acurate Neo ( tabela 3 ). Houve 23 procedimentos válvula-em-válvula, e 90 pacientes foram submetidos a pré-dilatação de válvula balão.

**Tabela 3:** Características do procedimento

Tipo de válvula	
CoreValve Evolut R	140 (41)
CoreValve Evolut Pro	72 (21)
Acurate Neo	44 (13)
SAPIEN 3	33 (10)
Portico	31 (9)
CoreValve	14 (4)
LOTUS	6 (2)
Pré-dilatação de balão	90 (27)
Válvula-em-válvula	23 (7)

*A tabela 3 apresenta as características do procedimento da amostra TAVR. Os valores foram apresentados como número de casos (%).*

### Distúrbios de condução pós-TAVR e preditores nos ECG

Depois da TAVR, 50,9% dos pacientes apresentaram novos distúrbios de condução ( tabela 4 ). Em relação à condução AV, 13,6% dos pacientes desenvolveram BAV de baixo grau (Mobitz I de 1º grau ou 2º grau) e 12,4% desenvolveram BAV avançado (Mobitz II de 2º grau ou 3º grau). Em relação à condução IV, o BRE de novo foi o distúrbio mais frequente (32,2%).

**Tabela 4:** Novos distúrbios de condução

N	172 (50,9)
**Condução AV**	
BAV de 1º grau	42 (12,4)
BAV Mobitz I de 2º grau	4 (1,2)
BAV Mobitz II de 2º grau	2 (0,6)
BAV de 3º grau	40 (11,8)
**Condução IV**	
Bloqueio fascicular	5 (1,5)
BRE	109 (32,2)
BRD	1 (0,3)
BRA	1 (0,3)
NICD	2 (0,6)

*A tabela 4 mostra distúrbios de condução de novo após o implante valvar. Os valores foram apresentados como número de casos (%). AV: atrioventricular; BAV: bloqueio atrioventricular; BRE: bloqueio de ramo esquerdo; BRD: bloqueio de ramo direito; BRA: bloqueio de ramo alternante; NICD: retardo de condução intraventricular não específico.*

**Figura 2:**
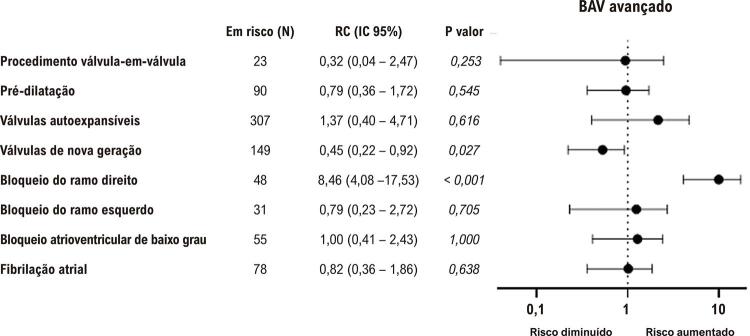
Preditores de BAV avançado. A figura 2 apresenta um gráfico de floresta que compila os principais preditores possíveis de BAV avançado. O teste qui-quadrado foi usado para analisar a diferença entre os grupos. BAV: bloqueio atrioventricular avançado.

A FA prévia não foi associada a implante de MPD ou BAV avançado. O BAV de baixo grau, quando comparado a pacientes com condução AV normal, foi associado a um índice de implante de MPD mais alto (30,4% vs. 16,2%, p=0,016), mas não a BAV avançado ( Figuras 2 e 3 ).

**Figura 3:**
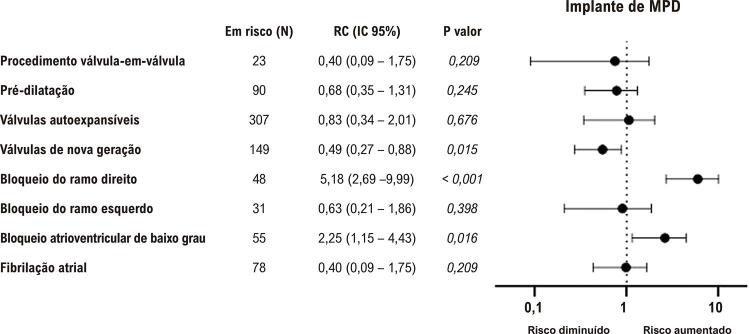
Preditores de implante de MPD. A figura 3 apresenta um gráfico de floresta que resume os principais preditores possíveis de implante de MPD. O teste qui-quadrado foi usado para analisar a diferença entre os grupos. MPD: marca-passo definitivo.

Em relação à condução IV, o BRE prévio não aumentou o risco de novo BAV avançado ou implante de MPD. Em contraste, a presenças de BRD prévio demonstrou ser um fator de risco forte para BAV avançado (7,2% vs. 39,6%, p<0,001) e implante de MPD (14,0% vs. 45,8%, p<0,001). O bloqueio fascicular e o NICD não foram associados a implante de MPD ou BAV avançado.

Três casos de BAV avançado foram revertidos logo após a TAVR (menos de 24h). Na alta hospitalar, 27,5% de BRE de novo foram resolvidos. Após 6 meses de acompanhamento, o índice de recuperação foi mais alto, com 56,1% dos casos revertidos à condução intraventricular normal.

### Procedimento TAVR e distúrbios de ritmo

**Figura 1:**
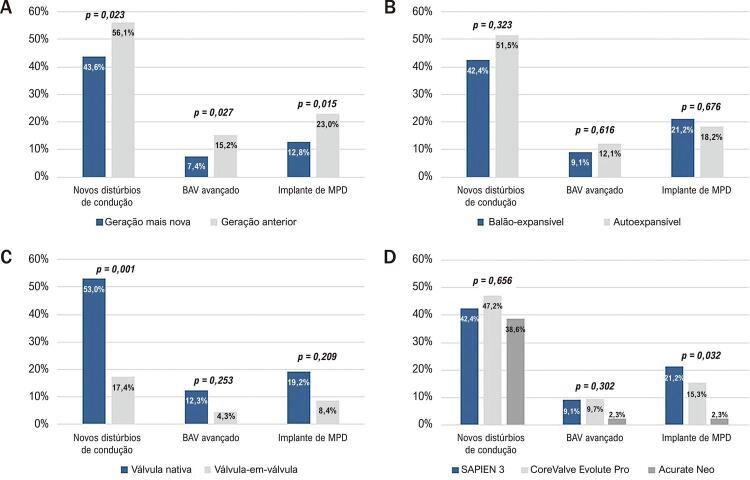
Procedimento TAVR e distúrbios de ritmo. A figura 1 apresenta a associação das características do procedimento com novos distúrbios de ritmo, BAV avançado e implante de MPD em relação à geração da válvula (A), válvula balão-expansível ou autoexpansível (B), implante válvula-em-válvula (C) ou modelos de válvulas de geração mais nova (D). BAV: bloqueio atrioventricular; MPD: marca-passo definitivo.

A maior proporção de novos distúrbios de condução foi observada com a válvula LOTUS (80% dos pacientes), seguida de Portico (71%), CoreValve (64%), CoreValve Evolut R (51%), CoreValve Evolut Pro (47%), SAPIEN 3 (42%) e Acurate Neo (39%). A tabela 5 e a figura 1 resumem os principais achados com base nas características dos procedimentos. Houve uma diferença significativa entre as válvulas de geração mais novas e as de geração mais antiga em relação à incidência de novos distúrbios de condução, BAV avançado e implante de MPD.

**Tabela 5:** Procedimento TAVR e distúrbios de ritmo

Procedimento	Novos distúrbios de ritmo	p valor	BAV avançado	p valor	Implante de MPD	p valor
**Válvulas de geração mais nova vs. anterior**		**0,023**		**0,027**		**0,015**
Geração mais nova	43,6%		7,4%		12,8%	
Geração anterior	56,1%		15,2%		23,0%	
**Válvulas balão-expansíveis vs. autoexpansíveis**		0,323		0,616		0,676
Balão-expansível	42,4%		9,1%		21,2%	
Autoexpansível	51,5%		12,1%		18,2%	
**Pré-dilatação**		0,320		0,545		0,245
Sem pré-dilatação	52,2%		12,4%		20,0%	
Pré-dilatação	46,1%		10,0%		14,4%	
**Válvula-em-válvula**		**0,001**		0,253		0,209
Válvula nativa	53,0%		12,3%		19,2%	
Válvula-em-válvula	17,4%		4,3%		8,7%	
**Válvulas de geração mais nova**		0,656		0,302		**0,032**
SAPIEN 3	42,4%		9,1%		21,2%	
CoreValve Evolut Pro	47,2%		9,7%		15,3%	
Acurate Neo	38,6%		2,3%		2,3%	

*A tabela 5 resume a associação das características do procedimento com novos distúrbios de ritmo, bloqueio atrioventricular (BAV) avançado e implante de marca-passo definitivo (MPD). Os dados foram apresentados em porcentagem e os p valores significativos, em negrito. Novos distúrbios de ritmo incluíram todos os distúrbios de condução de atrioventricular ou intraventricular de novo surgiram após o implante valvular aórtico percutâneo.*

A pré-dilatação não foi associada ao desenvolvimento de distúrbios de condução ou de diferenças na regressão desses distúrbios no acompanhamento de 6 meses. Ao se comparar válvulas balão-expansíveis e autoexpansíveis, também não foram encontradas diferenças estatisticamente significativas.

Procedimentos válvula-em-válvula foram associados a menos mudanças na condução, com apenas 17,4% dos pacientes desenvolvendo retardos de condução [RC=0,19 (95% IC 0,06-0,58)] e apenas 8,7% precisando de implante de MPD, apesar de um índice semelhante de distúrbios de condução AV e IV pré-TAVR.

Também foi realizada uma análise adicional incluindo apenas as válvulas de geração mais nova. Nesse grupo, não foram encontradas diferenças em distúrbios de condução novos e BAV avançado, mas uma diferença estatisticamente significativa foi encontrada em relação ao implante de MPD em favor da Acurate Neo (p=0,032).

### Implante de MPD e acompanhamento

No geral, 18,5% (N = 63) dos pacientes passaram por um implante de MPD após a TAVR, 81% de dispositivos de câmara dupla sem complicações importantes durante a admissão. O principal motivo para implante de marca-passo foi BAV avançado (60,3%), seguido de BRE com BAV de baixo grau (22,2%), BRE isolado (4,8%) e bloqueio de ramo alternante (BRA, 4,8%).

Na primeira avaliação do MPD, pacientes com BAV avançado apresentaram uma porcentagem mediana de EV de 80%, com 44,4% dos pacientes apresentando >90% de EV e 14,8% <1% de EV. Um ano após a TAVR, a porcentagem mediana de EV era de 83%, quase metade dos pacientes (46,2%) com EV >90% e 19,2% com EV abaixo de um por cento.

Em relação aos pacientes com BRE e BAV de baixo grau, a EV mediana na primeira avaliação foi 6% (44,4% tinham <1% de EV) e 11,1% tinham >90% de EV; a avaliação do marca-passo após um ano apresentou uma EV mediana de 2%, com metade dos pacientes apresentando EV abaixo de um por cento. A diferença de EV entre pacientes com BAV avançado e pacientes com BRE e BAV de baixo grau é estatisticamente significativa na primeira avaliação (p = 0,036). Depois de um ano após o implante de MPD, pacientes com BRE e BAV de baixo grau tendiam a ter EV (p = 0,062) e menos pacientes apresentaram EV >40% (33,3% vs. 73,1%, p = 0,065).

Em pacientes com BRE isolado ou BRA, a EV mediana foi de 9% e 13% na primeira avaliação, e 20% e 15% após um ano, respectivamente.

Os gráficos de floresta nas figuras 2 e 3 resumem as principais características com o novo surgimento de BAV avançado e implante de MPD nesta amostra.

## Discussão

Distúrbios de condução após TAVR continuam a ser um desafio e deve-se fazer um esforço para reconhecer os pacientes que estão em risco de defeitos de condução de alto grau e implante de MPD.

Neste estudo, entre 340 pacientes sem MPD prévio, metade apresentou novos distúrbios de condução após a TAVR, e 18,5% dos pacientes tiveram implante de MPD. De acordo com a literatura, o BRE de novo foi o distúrbio de condução mais frequente observado após o procedimento,^1^ ocorrendo em um terço dos pacientes.

Vários estudos identificaram distúrbios de condução pré-existentes (especificamente, bloqueio de AV de primeiro grau, BRD, BRE e bloqueio fascicular) como fatores de risco para implante de MPD após a TAVR.^1 , 2 , 5 , 11 , 12^ O papel do bloqueio de AV de primeiro grau como um fator de risco para distúrbios de condução é controverso em estudos recentes.^1 , 5 , 11 - 13^ Nesta amostra, encontrou-se uma relação significativa entre BAV de baixo grau prévio e implante de MPD (RC de 2,25), mas não com BAV avançado. Isso provavelmente pode ser explicado pelo fato de que um dos principais motivos para implante de MPD em nosso centro foi o BAV de baixo grau e BRE (22,2% dos implantes de MPD).

O BRD foi o único distúrbio no ECG pré-TAVR que foi associado a um aumento significativo no risco de BAV avançado e de implante de MPD, com um risco aproximadamente oito vezes maior de BAV avançado e cinco vezes maior de implante de MPD. Isso está de acordo com vários outros relatos que identificam o BRD como o fator de risco mais importante para BAV avançado/bloqueio cardíaco total e a necessidade de MPD após a TAVR.^1 , 7 , 12 - 14^ Na verdade, Watanabe et al. demonstraram que pacientes com BRD pré-existente, sem MPD, tinham um risco mais alto de morte cardíaca após a alta, levantando-se a hipótese de que isso poderia se dever ao desenvolvimento de BAV de alto grau.^15^

O BRE e o bloqueio fascicular anterior esquerdo são outros fatores de risco controversos para implante de MPD.^12 , 16^ Nossos achados não foram consistentes com essa hipótese, não mostrando relação com BAV avançado ou com implante de MPD.

As características do procedimento também estão implicadas na ocorrência de complicações de condução peri-TAVR. Vários relatos anteriores sugeriram índices mais altos de distúrbios de ritmo com pré-dilatação de válvula nativa e válvulas autoexpansíveis,^1 , 17 - 19^ embora isso não tivesse sido observado em nossa amostra, como sugerido por dados mais atuais.^20 , 21^ Procedimentos válvula-em-válvula foram associados e menos distúrbios de condução de novo (RC = 0,19) e essa diferença não foi explicada por diferenças estatisticamente significativas na condução AV ou IV pré-TAVR, de acordo com dados publicados anteriormente.^22^

Conforme proposto na análise sistemática,^23^ válvulas de nova geração foram associadas a uma incidência significativamente mais baixa de novos distúrbios de condução, BAV avançado e implante de MPD. Realizamos uma análise adicional incluindo apenas válvulas de geração mais nova, encontrando uma diferença estatisticamente significativa no implante de MPD em favor da válvula Acurate Neo, possivelmente explicada por uma força radial mais baixa que causa menos lesão mecânica.^24^ Em relação ao novo surgimento de distúrbios de condução, apenas três casos de BAV avançado (7%) foram revertidos durante a internação hospitalar, todos durante as primeiras 24 horas. Esses pacientes tiveram alta e apresentaram BAV avançado durante o acompanhamento. Em relação ao BRE, de acordo com dados publicados,^2 , 6 , 13 , 25^ uma porcentagem mais alta dos casos foi revertida, com mais de um quarto dos casos sendo resolvidos antes da alta hospitalar e mais da metade após 6 meses de acompanhamento.

O BRE de novo continua sendo o distúrbio de ritmo mais desafiador para se lidar pós-TAVR. De acordo com os relatos anteriores, alguns pacientes com novo surgimento de BRE desenvolvem BAV avançado.^2 , 7 , 26^ mas uma proporção significativa terá seu ECG parcial ou totalmente normalizado^1 , 5 , 6 , 8^ Embora dados atuais não justifiquem o implante sistemático de MPD nesses pacientes, alguns estudos sugeriram risco mais alto de BAV avançado retardado durante o acompanhamento em pacientes com QRS longo (mais de 150 - 160 ms), especialmente quando associado a um intervalo PR longo (mais de 240 ms). De acordo com o recente consenso do JACC Scientific Expert Panel, pode ser razoável implantar MPD em pacientes com BRE e um intervalo PR acima de 240 ms ou BRE com duração de QRS maior que 150 - 160 ms.^1^ O consenso de especialistas da ACC de 2020 também considera a possibilidade de estudo eletrofisiológico e recomenda o monitoramento ambulatorial do ritmo por, no mínimo, 14 dias após a alta, com um monitor capaz de comunicar episódios de BAV avançado, permitindo a ativação imediata de serviços médicos emergenciais.^5^

Realizamos uma análise independente em pacientes com MPD de novo demonstrando que pacientes que tiveram MPD devido a BAV avançado tinham uma porcentagem mais alta de EV do que os pacientes que receberam MPD por outras indicações, com 44,4% e 46,2% apresentando mais de 90% de EV na primeira avaliação do MPD e um ano após o implante, respectivamente. Esses resultados são consistentes com um estudo publicado recentemente na Itália.^27^ No subgrupo dos pacientes com implantes de MPD devido a BRE e BAV de baixo grau, a EV mediana foi muito baixa (2% após um ano), com metade dos pacientes com menos de 1% de EV, e apenas 33,3%, mais de 40%. Apesar dessa porcentagem de EV mais baixa, não se pode excluir o uso de estimulação durante episódios paroxísticos de bradicardia extrema ou BAV avançado. Esses resultados melhoram o conhecimento em relação à dependência do MPD de longo prazo em pacientes pós-TAVR, destacando uma seleção mais precisa de pacientes com BRE que beneficie um implante de MPD e fortalecendo a importância do monitoramento ambulatorial do ritmo em pacientes com BRE novo para reconhecer eventos de BAV avançado imediatamente. Entretanto, a EV alta observada em pacientes com BAV avançado reforça a justificativa de se implantar mais modos estimulação, tais como, a estimulação do ramo His ou estimulação biventricular nesses pacientes.

### Limitações

O presente estudo foi um estudo observacional retrospectivo de centro único, que foi sua principal limitação. Embora todos os ECG tenham sido avaliados por cardiologistas, não houve Core Lab responsável por sua revisão. As durações dos intervalos PR e QRS não foram registradas.

## Conclusões

Nosso estudo demonstrou que o BRE foi o distúrbio de condução de novo mais frequente após a TAVR, com mais da metade dos casos se resolvendo nos primeiros 6 meses. O BRD prévio e o BAV de baixo grau foram significativamente associados a índices mais altos de implante de MPD pós-TAVR, com um aumento de cinco vezes do risco em pacientes com BRD. Diferentemente da pré-dilatação da válvula nativa e válvulas autoexpansíveis, observou-se que os procedimentos válvula-em-válvula estavam associados a significativamente menos distúrbios de condução, e a válvula Acurate Neo estava associada a menos implantes de MPD. Em relação ao acompanhamento do MPD, pacientes que tinham um MPD devido a BAV avançado tiveram uma porcentagem significativamente mais alta de EV do que pacientes que receberam o MPD por outros motivos, tais como BRE e BAV de baixo grau. No geral, esse relato destaca a importância de mais evidências para selecionar, de maneira mais precisa, pacientes com BRE que podem se beneficiar do implante de MPD e os que não podem, fortalecendo a estratégia de monitoramento ambulatorial próximo para reconhecer imediatamente eventos de BAV avançado nesses pacientes. Além disso, os resultados de pacientes com BAV avançado reforçam a justificativa de se implantar modos estimulação mais fisiológicos nesse grupo.

## References

[1] Rodes-Cabau J, Ellenbogen KA, Krahn AD, Latib A, Mack M, Mittal S (2019). Management of Conduction Disturbances Associated With Transcatheter Aortic Valve Replacement: JACC Scientific Expert Panel. J Am Coll Cardiol.

[2] Auffret V, Puri R, Urena M, Chamandi C, Rodrigues-Gabella T, Phillipon F (2017). Conduction Disturbances After Transcatheter Aortic Valve Replacement: Current Status and Future Perspectives. Circulation.

[3] Siontis GCM, Overtchouk P, Cahill TJ, Modine T, Prendergast B, Praz F (2019). Transcatheter aortic valve implantation vs. surgical aortic valve replacement for treatment of symptomatic severe aortic stenosis: an updated meta-analysis. Eur Heart J.

[4] Bagur R, Rodes-Cabau J, Gurvitch R, Dumont É, Velianou JL, Manazzoni J (2012). Need for permanent pacemaker as a complication of transcatheter aortic valve implantation and surgical aortic valve replacement in elderly patients with severe aortic stenosis and similar baseline electrocardiographic findings. JACC Cardiovasc Interv.

[5] Lilly SM, Deshmukh AJ, Epstein AE, Ricciardi MJ, Sheenivas S, Vilagapudi P (2020). 2020 ACC Expert Consensus Decision Pathway on Management of Conduction Disturbances in Patients Undergoing Transcatheter Aortic Valve Replacement. J Am Coll Cardiol.

[6] Rodes-Cabau J, Urena M, Nombela-Franco L, Amat-Santos I, Kleiman N, Munozz-Garcia M, Atienza F (2018). Arrhythmic Burden as Determined by Ambulatory Continuous Cardiac Monitoring in Patients With New-Onset Persistent Left Bundle Branch Block Following Transcatheter Aortic Valve Replacement: The MARE Study. JACC Cardiovasc Interv.

[7] Toggweiler S, Stortecky S, Holy E, Zuk K, Cuculi F, Nietlispach F (2016). The Electrocardiogram After Transcatheter Aortic Valve Replacement Determines the Risk for Post-Procedural High-Degree AV Block and the Need for Telemetry Monitoring. JACC Cardiovasc Interv.

[8] Jorgensen TH, De Backer O, Gerds TA, Bieliauskas G, Svendsen JH, Sondergaard L (2018). Immediate Post-Procedural 12-Lead Electrocardiography as Predictor of Late Conduction Defects After Transcatheter Aortic Valve Replacement. JACC Cardiovasc Interv.

[9] Kappetein AP, Head SJ, Genereux P, Piazza N, van Mieghem NM (2012). Updated standardized endpoint definitions for transcatheter aortic valve implantation: the Valve Academic Research Consortium-2 consensus document. Eur Heart J.

[10] Kusumoto FM, Schoenfeld MH, Barrett C, Edgerton JR, Ellenbogen KA, Gold MR (2019). 2018 ACC/AHA/HRS Guideline on the Evaluation and Management of Patients With Bradycardia and Cardiac Conduction Delay: A Report of the American College of Cardiology/American Heart Association Task Force on Clinical Practice Guidelines and the Heart Rhythm Society. Circulation.

[11] Mangieri A, Lanzillo G, Bertoldi L, Jabbour RJ, Regazzoli D, Ancona MB (2018). Predictors of Advanced Conduction Disturbances Requiring a Late (>/=48 H) Permanent Pacemaker Following Transcatheter Aortic Valve Replacement. JACC Cardiovasc Interv.

[12] Siontis GC, Juni P, Pilgrim T, Stortecky S, Büllesfeld L, Meier B (2014). Predictors of permanent pacemaker implantation in patients with severe aortic stenosis undergoing TAVR: a meta-analysis. J Am Coll Cardiol.

[13] Nazif TM, Dizon JM, Hahn RT, Xu K, Babaliaros V, Douglas PS (2015). Predictors and clinical outcomes of permanent pacemaker implantation after transcatheter aortic valve replacement: the PARTNER (Placement of AoRtic TraNscathetER Valves) trial and registry. JACC Cardiovasc Interv.

[14] Mack MJ, Leon MB, Thourani VH, Makkar R, Kodali SK, Russo M (2019). Transcatheter Aortic-Valve Replacement with a Balloon-Expandable Valve in Low-Risk Patients. N Engl J Med.

[15] Watanabe Y, Kozuma K, Hioki H, Kawashima H, Nara Y, Kataoka A (2016). Pre-Existing Right Bundle Branch Block Increases Risk for Death After Transcatheter Aortic Valve Replacement With a Balloon-Expandable Valve. JACC Cardiovasc Interv.

[16] Fischer Q, Himbert D, Webb JG, Eltchaninoff H, Muñoz-García AJ, Tamburino C (2018). Impact of Preexisting Left Bundle Branch Block in Transcatheter Aortic Valve Replacement Recipients. Circ Cardiovasc Interv.

[17] Campelo-Parada F, Nombela-Franco L, Urena M, Regueiro A, Jiménez-Quevedo P, Del Trigo M (2018). Timing of Onset and Outcome of New Conduction Abnormalities Following Transcatheter Aortic Valve Implantation: Role of Balloon Aortic Valvuloplasty. Rev Esp Cardiol. (Engl Ed).

[18] Gensas CS, Caixeta A, Siqueira D, Sarmento -Leite G, Mangione J (2014). Predictors of permanent pacemaker requirement after transcatheter aortic valve implantation: insights from a Brazilian registry. Int J Cardiol.

[19] Banerjee K, Kandregula K, Sankaramangalam K, Anumandla A, Kumar A, Parikh P (2018). Meta-analysis of the Impact of Avoiding Balloon Predilation in Transcatheter Aortic Valve Implantation. Am J Cardiol.

[20] Thiele H, Kurz T, Feistritzer HJ, Stachel G, Hartung P, Eitel I (2020). Comparison of newer generation self-expandable vs. balloon-expandable valves in transcatheter aortic valve implantation: the randomized SOLVE-TAVI trial. Eur Heart J.

[21] Pagnesi M, Kim WK, Conradi L (2020). Impact of Predilatation Prior to Transcatheter Aortic Valve Implantation With the Self-Expanding Acurate neo Device (from the Multicenter NEOPRO Registry). Am J Cardiol.

[22] Ferrari E, Stortecky S, Heg D, Muller O, Nietlispach F, Tueller D (2019). The hospital results and 1-year outcomes of transcatheter aortic valve-in-valve procedures and transcatheter aortic valve implantations .in the native valves: the results from the Swiss-TAVI Registry. Eur J Cardiothorac Surg.

[23] van Rosendael PJ, Delgado V, Bax JJ (2018). Pacemaker implantation rate after transcatheter aortic valve implantation with early and new-generation devices: a systematic review. Eur Heart J.

[24] Okuno T, Lanz J, Pilgrim T (2020). ACURATE neo: How Is This TAVR Valve Doing to Fit into an Increasingly Crowded Field?. Curr Cardiol Rep.

[25] Houthuizen P, van der Boon RM, Urena M, Van Mieghem N, Brueren GB, Poels TT (2014). Occurrence, fate and consequences of ventricular conduction abnormalities after transcatheter aortic valve implantation. EuroIntervention.

[26] Akin I, Kische S, Paranskaya L, Schneider H, Rehders TC, Trautwauin U (2012). Predictive factors for pacemaker requirement after transcatheter aortic valve implantation. BMC Cardiovasc Disord.

[27] Baldi E, Compagnone M, Errigo D, Ferlini M, Ziachi M, Castagno D (2020). Long-term percentage of ventricular pacing in patients requiring pacemaker implantation after transcatheter aortic valve replacement: A multicenter 10-year experience. Heart Rhythm.

