# Supplementary material for: Dependência de Pacing a Longo-Prazo e Preditores de Implante de Pacemaker após Implante Percutâneo de Prótese Valvular Aórtica – 1 Ano de Seguimento
Source: Arq Bras Cardiol. 2022 Jul 13;119(4):522–30. [Article in Portuguese] doi: 10.36660/abc.20210613 (PMC9563875; doi:10.36660/abc.20210613)
Supplement: Translation

# Translation: Long-Term Ventricular Pacing Dependency and Pacemaker Implantation Predictors after Transcatheter Aortic Valve Replacement – A 1-Year Follow-Up

**Authors:** Ricardo Alves Pinto, Tânia Proença, Miguel Martins Carvalho, Gonçalo Pestana, Ana Lebreiro, Luis Adão, Filipe Macedo

**Affiliations:** 1 Portugal Departamento de Cardiologia, Centro Hospitalar Universitário de São João, E.P.E., Porto – Portugal; 2 Portugal Faculdade de Medicina da Universidade do Porto, Porto – Portugal

**Keywords:** Aortic Valve Stenosis, Atrioventriclar Block, Transcatheter Aortic Valve Replacement, Pacemaker,Artificial, Heart Valve Prosthesis Implantation, Cardiac Conduction System Disease

## Abstract

**Background:**

Conduction disturbances (CD) are the most frequent complication after transcatheter aortic valve replacement (TAVR), and there continues to be a lack of consensus on their management.

**Objective:**

To assess new CD and permanent pacemaker (PPM) implantation after TAVR and to evaluate the ventricular pacing percentage (VP) up to 1 year of follow-up.

**Methods:**

Patients who underwent TAVR from October 2014 to November 2019 were enrolled; patients with previous PPM were excluded. Clinical, procedure, ECG, and PPM data were collected up to 1 year after implantation. The significance level adopted in the statistical analysis was 0.05.

**Results:**

A total of 340 patients underwent TAVR. The most frequent CD was the new left bundle branch block (LBBB; 32.2%), which 56% resolved after 6 months. Right bundle branch block (RBBB) was the biggest risk factor for advanced atrioventricular block (AVB) [OR=8.46; p<0.001] and PPM implantation [OR=5.18, p<0.001], followed by previous low-grade AVB [OR=2.25; p=0.016 for PPM implantation]. Regarding procedure characteristics, newer generation valves and valve-in-valve procedures were associated with fewer CDs. Overall, 18.5% of patients had a PPM implanted post-TAVR. At first PPM evaluation, patients with advanced AVB had a median percentage of VP of 80% and 83% at one year. Regarding patients with LBBB plus low-grade AVB, median VP was lower (6% at first assessment, p=0.036; 2% at one year, p = 0.065).

**Conclusion:**

LBBB was the most frequent CD after TAVR, with more than half being resolved in the first six months. RBBB was the major risk factor for advanced AVB and PPM implantation. Advanced AVB was associated with a higher percentage of VP at 1 year of follow-up.

## Introduction

Transcatheter aortic valve replacement (TAVR) is a well-established procedure to treat patients with symptomatic severe aortic stenosis at increased or prohibitive surgical risk. Increased experience has led to a growing consideration of TAVR as an option to people at lower risk.^1 - 3^ The widespread adoption of TAVR was accompanied by a reduction in the majority of periprocedural complications, except for new conduction disturbances and consequent need for PPM implantation.^1 , 4 , 5^ New LBBB, with an incidence of about 25% (4% to 65%), is the most frequently documented rhythm disorder after TAVR and probably the most challenging.^1^ Although often self-limited, a significant percentage of these patients evolve to advanced AVB or complete heart block, the most serious complications of conduction after-TAVR.^1 , 2 , 4 , 6 , 7^

Major questions remain about the management of conduction disturbances after TAVR, leading to distinct approaches among different centers. Patients commonly continue to be monitored with telemetry and daily electrocardiogram (ECG) after the procedure, sometimes with backup temporary pacemaker, increasing the hospitalization length and procedural cost.^4 , 7 , 8^ There are limited data on risk factors for the development of advanced AVB and the need to maintain a temporary pacemaker, which also translates into varying rates of PPM implantation post-TAVR.^1 , 7^

The aim of the present study was to describe new conduction disturbances and PPM implantation in patients undergoing TAVR with either a balloon-expandable or a self-expandable valve prosthesis. We also evaluated the percentage of VP in patients who underwent PPM implantation up to 1 year of follow-up.

## Methods

### Study population

The present study included a sample of consecutive patients undergoing TAVR at Centro Hospitalar Universitário de São João, E.P.E., a tertiary center in Porto, Portugal, from October 2014 to November 2019 (n = 371). Patients who had PPM previous to valve implantation were excluded (n = 31). The remaining 340 patients were retrospectively analyzed. Clinical, electrocardiographic, echocardiographic, and procedure data were collected at presentation and up to 1 year after implantation, including systematic interrogation of implanted PPM. This study was approved by the institutional ethics committee.

### Definitions, data, and ECG collection

Clinical endpoints and definition of conduction disturbances were in accordance with the Valve Academic Research Consortium (VARC)-2 Consensus and the consensus by JACC Scientific Expert Panel, respectively.^1 , 9^ ECGs were systematically obtained at baseline (usually the day before TAVR), immediately after valve implantation (at admission in cardiac care unit) and at least daily until hospital discharge. All patients had continuous electrocardiographic monitoring during hospital stay. Most ECGs in our institution were electronically recorded, and were assessed and reviewed by cardiologists. Clinical, echocardiographic, and procedure data were collected from digital records. Low grade AVB was defined as 1^st^degree or 2^nd^degree Mobitz I AVB. Advanced AVB was defined as 2^nd^degree Mobitz II or 3^rd^degree AVB.

### Procedure

Patients submitted to TAVR with self-expandable (Medtronic CoreValve, Medtronic CoreValve Evolut R, Medtronic CoreValve Evolut Pro, Boston Scientific Acurate Neo, Abbott Portico, and Boston Scientific LOTUS) and with balloon-expandable (Edwards SAPIEN 3) valves were included. All patients had a temporary transvenous pacing catheter placed in the right ventricle. Depending on new-onset conduction disturbances or pre-procedure risk of rhythm disorder, and in accordance with the consensus by JACC Scientific Expert Panel,^1^ the temporary pacemaker was removed, either immediately in the catheterization laboratory or later during hospitalization (usually 24 – 48h). For the purpose of this study, the newer generation valve analysis included procedures with SAPIEN 3, CoreValve Evolut Pro and Acurate Neo valves, while the remaining were classified as earlier generation valves.

### Permanent pacemaker indication and follow-up

PPM were implanted according to 2018 ACC/AHA/HRS guidelines for bradycardia and cardiac conduction delay and in accordance with JACC Scientific Expert Panel.^1 , 10^ All devices were reviewed on day 1 and 7 after implantation. Intrinsic AV conduction was systematically queried and algorithms to minimize VP were applied (Managed Ventricular Pacing mode or AAI mode with backup VVI pacing in most patients). For the purpose of the study, first PPM evaluation was defined as first ambulatory device evaluation after discharge (median time 3 months after implantation, IQR 3 - 4 months) and one-year evaluation was defined as second ambulatory device evaluation (median time 12 months after implantation, IQR 10 - 12 months). Because some patients were followed up at other medical institutions, data from PPM follow-up was unavailable in 30% and 43% of patients for first PPM and one-year evaluations, respectively.

### Statistical analysis

Data are presented as median (interquartile range [IQR]) for continuous variables, and as number and percentages for categorical variables. One-sample Kolmogorov-Smirnov test was performed to evaluate normal distribution. Categorical variables were compared using the chi-square test; odds ratios (OR) are presented when considered relevant. Continuous variables were compared using the Mann-Whitney U test. Differences were considered statistically significant when p value < 0.05. Statistical analysis was performed in IBM SPSS Statistics version 25.

## Results

### Study population

A total of 340 patients undergoing TAVR between October 2014 and November 2019 were included in our sample, after excluding 31 patients with a previous PPM.

Baseline characteristics of the study sample are summarized in table 1 and table 2 . Median age was 81 years (IQR 76 to 85 years) and 57% of the patients were female.

**Table 1:** Baseline

N	340
Age, yrs (IQR)	81 (76 - 81)
Female (%)	193 (57)
Hypertension (%)	294 (87)
Diabetes (%)	127 (37)
Dyslipidemia (%)	244 (72)
Prior kidney disease (%)	185 (62)
on dialysis (%)	10 (3)
Atrial fibrillation (%)	78 (23)
Preserved LV function (%)	244 (73)
Bicuspid valve (%)	8 (3)
Aortic valve area (IQR)	0,7 cm^2^(0,6 – 0,9)
Transvalvular pressure gradient (IQR)	46 mmHg (39.5 - 59)
LV ejection fraction (IQR)	60 % (44 - 65)
Severe aortic regurgitation (%)	15 (6)

*Table 1 presents the baseline characteristics of study population. Values were presented as median (IQR) or number of cases (%). IQR: interquartile range; yrs: years-old; LV: left ventricle.*

**Table 2:** Pre-TAVR rhythm characteristics

Rhythm	
Sinus rhythm	262 (77)
Atrial Fibrillation	78 (23)
**AV conduction**	
Normal AV conduction	207 (79)
1st degree AVB	53 (20)
2nd degree Mobitz I AVB	2 (1)
**IV conduction**	
LBBB	31 (9)
RBBB	25 (7)
Left anterior fascicular block	24 (7)
Bifascicular block	23 (7)
Nonspecific intraventricular conduction delay	33 (10)

*Table 2 summarizes cardiac rhythm, atrioventricular (AV) conduction and intraventricular (IV) conduction of study population before TAVR. AV conduction was considered only in sinus rhythm. Values were presented as number of cases (%). LBBB: left bundle branch block; RBBB: right bundle branch block; AVB: atrioventricular block.*

At baseline, 77% of patients were in sinus rhythm and 23% AF; in patients who were in sinus rhythm (SR), most had normal atrioventricular (AV) conduction. Regarding intraventricular (IV) conduction, 60% had no conduction disturbance, and the most frequent disturbance was nonspecific intraventricular conduction delay (NICD; table 2 ).

Self-expandable CoreValve Evolut R was the most frequently used valve (41% of cases), followed by CoreValve Evolut Pro and Acurate Neo ( Table 3 ). There were 23 valve-in-valve procedures, and 90 patients underwent balloon valve pre-dilation.

**Table 3:** Procedure characteristics

Valve type	
CoreValve Evolut R	140 (41)
CoreValve Evolut Pro	72 (21)
Acurate Neo	44 (13)
SAPIEN 3	33 (10)
Portico	31 (9)
CoreValve	14 (4)
LOTUS	6 (2)
Balloon pre-dilation	90 (27)
Valve-in-valve	23 (7)

*Table 3 shows the procedure characteristics of the TAVR sample. Values were presented as number of cases (%).*

### Conduction Disturbances Post-TAVR and ECG predictors

After TAVR, 50.9% of the patients exhibited new conduction disturbances ( table 4 ). Regarding AV conduction, 13.6% of patients developed low grade AVB (1^st^degree or 2^nd^degree Mobitz I) and 12.4% developed advanced AVB (2^nd^degree Mobitz II or 3^rd^degree). Regarding IV conduction, de novo LBBB was the most frequent disturbance (32.2%).

**Table 4:** New conduction disturbances

N	172 (50.9)
**AV conduction**	
1st degree AVB	42 (12.4)
2nd degree Mobitz I AVB	4 (1.2)
2nd degree Mobitz II AVB	2 (0.6)
3rd degree AVB	40 (11.8)
**IV conduction**	
Fascicular block	5 (1.5)
LBBB	109 (32.2)
RBBB	1 (0.3)
ABBB	1 (0.3)
NICD	2 (0.6)

*Table 4 shows de novo conduction disturbances after valve implantation. Values were presented as number of cases (%). AV: atrioventricular; AVB: atrioventricular block; LBBB: left bundle branch block; RBBB: right bundle branch block; ABBB: alternating bundle branch block; NICD: nonspecific intraventricular conduction delay.*

Previous AF was not associated with advanced AVB or PPM implantation. Low-grade AVB, when compared with patients with normal AV conduction, was associated with a higher PPM implantation rate (30.4% vs 16.2%, p=0.016), but not with advanced AVB ( Figures 2 and 3 ).

**Figure 2:** Predictors of advanced AVB. Figure 2 showed a forest plot that compiled the main possible predictors of advanced AVB. Chi-square test was used to analyze the difference between groups. AVB: atrioventricular block.

**Figure 3:** Predictors of PPM implantation. Figure 3 displayed a forest plot that summarized the main possible predictors of PPM implantation. Chi-square test was used to analyze the difference between groups. PPM: permanent pacemaker.

Concerning IV conduction, previous LBBB did not increase the risk of new advanced AVB or PPM implantation. By contrast, the presence of previous RBBB proved to be a strong risk factor for advanced AVB (7.2% vs 39.6%, p<0.001) and PPM implantation (14.0% vs 45.8%, p<0.001). Fascicular block and NICD were not associated with advanced AVB or PPM implantation.

Three cases of advanced AVB reverted early after TAVR (less than 24h). Upon hospital discharge, 27.5% of de novo LBBB was resolved. After 6 months of follow-up, the rate of recovery was higher, with 56.1% of the cases reverted to normal intraventricular conduction.

### TAVR procedure and rhythm disturbances

The highest proportion of new conduction disturbances was seen with the LOTUS valve (80% of patients), followed by Portico (71%), CoreValve (64%), CoreValve Evolut R (51%), CoreValve Evolut Pro (47%), SAPIEN 3 (42%), and Acurate Neo (39%). Table 5 and Figure 1 summarize the main findings based on procedure characteristics. There was a significant difference between newer and earlier generation valves regarding incidence of new conduction disturbances, advanced AVB and PPM implantation.

**Table 5:** TAVR procedure and rhythm disturbances

Procedure	New rhythm disturbances	p-value	Advanced AVB	p-value	PPM implantation	p-value
**Newer vs earlier generation valves**		**0.023**		**0.027**		**0.015**
Newer generation	43.6%		7.4%		12.8%	
Earlier generation	56.1%		15.2%		23.0%	
**Balloon- vs self-expandable valves**		0.323		0.616		0.676
Balloon-expandable	42.4%		9.1%		21.2%	
Self-expandable	51.5%		12.1%		18.2%	
**Pre-dilation**		0.320		0.545		0.245
No pre-dilation	52.2%		12.4%		20.0%	
Pre-dilation	46.1%		10.0%		14.4%	
**Valve-in-valve**		**0.001**		0.253		0.209
Native valve	53.0%		12.3%		19.2%	
Valve-in-valve	17.4%		4.3%		8.7%	
**Newer generation valves**		0.656		0.302		**0.032**
SAPIEN 3	42.4%		9.1%		21.2%	
CoreValve Evolut Pro	47.2%		9.7%		15.3%	
Acurate Neo	38.6%		2.3%		2.3%	

*Table 5 summarizes the association of procedure characteristics with new rhythm disturbances, advanced atrioventricular block (AVB), and permanent pacemaker (PPM) implantation. Data were presented in percentage and significative p-values in bold. New rhythm disturbances included any de novo atrioventricular or intraventricular conduction disturbance that appeared after transcatheter aortic valve implantion.*

**Figure 1:** TAVR procedure and rhythm disturbances. Figure 1 displayed the association of procedure characteristics with new rhythm disturbances, advanced AVB, and PPM implantation concerning valve generation (A), balloon- or self-expandable valve (B), valve-in-valve implantation (C), or newer generation valve model (D). AVB: atrioventricular block; PPM: permanent pacemaker.

Pre-dilation was not associated with development of conduction disorders nor differences in regression of these disturbances upon 6 months of follow-up. When comparing balloon-expandable with self-expandable valves, no statistically significant difference was found.

Valve-in-valve procedures were associated with fewer changes in conduction, with only 17.4% of patients developing conduction delays [OR=0.19 (95% CI 0.06-0.58)] and only 8.7% requiring PPM implantation, despite a similar rate of pre-TAVR AV and IV conduction disturbances.

An additional analysis was also conducted, including only newer generation valves. In this group, no difference was found in new conduction disturbances and advanced AVB, but a statistically significant difference was found in PPM implantation in favor of Acurate Neo (p=0.032).

### PPM implantation and follow-up

Overall, 18.5% (N = 63) had a PPM implanted after TAVR, 81% dual-chamber devices, and no major complications occurred during admission. The main reason for pacemaker implantation was advanced AVB (60.3%), followed by LBBB with low-grade AVB (22.2%), isolated LBBB (4.8%), and alternating bundle branch block (ABBB, 4.8%).

Upon first PPM evaluation, patients with advanced AVB had a median percentage of VP of 80%, with 44.4% of patients presenting >90% of VP and 14.8% <1% of VP; one year after TAVR the median percentage of VP was 83%, almost half of patients (46.2%) with VP >90% and 19.2% with VP under one percent.

Regarding patients with LBBB plus low-grade AVB, median VP upon first assessment was 6% (44.4% had < 1% of VP) and 11.1% had >90% of VP; PM evaluation at one year showed a median VP of 2%, half of patients with VP under one percent. The difference in VP between patients with advanced AVB and patients with LBBB plus low-grade AVB is statistically significant in the first evaluation (p = 0.036). After one year of PPM implantation, patients with LBBB plus low-grade AVB tended to have lower VP (p = 0.062) and lesser patients with VP >40% (33.3% vs 73.1%, p = 0.065).

In patients with isolated LBBB or ABBB, median VP was 9% and 13% at the first evaluation, and 20% and 15% after one year, respectively.

The forest plots in Figures 2 and 3 summarize the main characteristics associated with new-onset advanced AVB and PPM implantation in our sample.

## Discussion

Conduction disturbances after TAVR continue to be challenging, and an effort should be made to recognize patients at risk for high-degree conduction defects and PPM implantation.

In the present study study, among 340 patients without previous PPM, half exhibited new conduction disturbances after TAVR, and 18.5% of patients had a PPM implanted. In accordance with literature, de novo LBBB was the most frequent conduction disturbance observed post-procedure,^1^ occurring in one-third of the patients.

Several studies have identified pre-existing conduction disturbances (namely first-degree AV block, RBBB, LBBB, and fascicular block) as risk factors for PPM implantation after TAVR.^1 , 2 , 5 , 11 , 12^ The role of first-degree AV block as a risk factor for conduction disturbance has proven to be controversial in recent studies.^1 , 5 , 11 - 13^ In our sample, a significant relation between previous low-grade AVB and PPM implantation (OR of 2.25) was found, but not with advanced AVB. This can most likely be explained by the fact that one of the main reasons to implant a PPM in our center was low-grade AVB plus LBBB (22.2% of PPM implantations).

RBBB was the only disturbance in pre-TAVR ECG that was associated with a significant increase in the risk of both advanced AVB and PPM implantation, with an approximately eightfold increased risk of advanced AVB and five times more risk of PPM implantation. This is in agreement with several other reports that identified RBBB as the most important risk factor for advanced AVB / complete heart block and need for PPM following TAVR.^1 , 7 , 12 - 14^ In fact, Watanabe et al. demonstrated that patients with pre-existing RBBB, without PPM, had a higher risk for cardiac death after discharge, hypothesizing this could be due to the development of high-grade AVB.^15^

LBBB and left anterior fascicular block are other controversial risk factors for PPM implantation.^12 , 16^ Our findings were not consistent with that hypothesis, showing no relation with more advanced AVB nor PPM implantation.

Procedure characteristics are also implicated in the occurrence of peri-TAVR conduction complications. Several earlier reports suggested higher rates of rhythm disorders with native valve pre-dilation and self-expandable valves,^1 , 17 - 19^ although this was not observed in our sample, as has been suggested by more contemporary data.^20 , 21^ Valve-in-valve procedures were associated with less de novo conduction disturbances (OR = 0.19), and this difference was not explained by statistically significant differences in pre-TAVR AV or IV conduction, which runs in line with previously published data.^22^

As proposed in a systematic review,^23^ newer generation valves were associated with a significantly lower incidence of new conduction disturbances, advanced AVB, and PPM implantation. An additional analysis was conducted, including only newer generation valves, finding a statistically significant difference in PPM implantation in favor of Acurate Neo, possibly explained by a lower radial force causing less mechanical injury.^24^ Regarding new-onset conduction disturbances, only three cases of advanced AVB (7%) reverted during hospitalization, all during the first 24 hours; these were discharged and presented no advanced AVB during follow-up. Regarding LBBB, in accordance with published data,^2 , 6 , 13 , 25^ a higher percentage of cases were reverted, with more than a quarter being resolved before hospital discharge and more than half after 6 months of follow-up.

De novo LBBB remains the most challenging rhythm disorder to handle post-TAVR. According to previous reports, some patients with new-onset LBBB will develop advanced AVB,^2 , 7 , 26^ but a significant proportion will partially or completely normalize their ECG.^1 , 5 , 6 , 8^ Although current data do not support systematic implantation of PPM in these patients, some studies have suggested a higher risk of delayed advanced AVB during follow-up in patients with long QRS (over 150 - 160 ms), particularly when associated with a long PR interval (more than 240 ms). According to the recent JACC Scientific Expert Panel’s consensus, it may be reasonable to implant PPM in patients with LBBB and a PR interval over 240 ms or LBBB with QRS duration more than 150 - 160 ms.^1^ The 2020 ACC expert consensus also considers the possibility of electrophysiological study and recommends ambulatory rhythm monitoring for at least 14 days after hospital discharge with a monitor capable of communicating episodes of advanced AVB, allowing prompt activation of emergency medical services.^5^

This study conducted an independent analysis in patients with de novo PPM, showing that patients who had a PPM due to advanced AVB had a higher percentage of VP than patients receiving a PPM for other indications, with 44.4% and 46.2% presenting more than 90% of VP uopn first PPM evaluation and one year after implantation, respectively; these results are consistent with a recently published study from Italy.^27^ In the subgroup of patients implanting PPM due to LBBB plus low-grade AVB, the median VP was very low (2% at one year), with half having less than 1% of VP and only 33.3% more than 40%. Despite this lower percentage of VP, one cannot exclude pacing use during brief paroxysmal episodes of extreme bradycardia or advanced AVB. These results enhance the knowledge regarding PPM long-term dependency in post-TAVR patients, highlighting a more accurate selection of LBBB patients that benefit from PPM implantation and strengthening the importance of ambulatory rhythm monitoring in new-LBBB patients to promptly recognize advanced AVB events. On the other hand, high VP observed in patients with advanced AVB reinforces the rationale of implanting more physiological modes of pacing like His bundle pacing or biventricular pacing in these patients.

### Limitations

The present study was a single-center retrospective observational study, which was its major limitation. Although ECGs were all assessed by cardiologists, there was no Core Lab responsible for ECG revision. PR and QRS interval durations were not recorded.

## Conclusions

This study showed that LBBB was the most frequent de novo conduction disturbance after TAVR, with more than half of the cases being resolved in the first 6 months. Previous RBBB and low-grade AVB were significantly associated with a higher rate of PPM implantation post-TAVR, with a fivefold increase of risk in patients with RBBB. Unlike native valve pre-dilation and self-expandable valves, valve-in-valve procedures were related to significantly less conduction disturbances, and the Acurate Neo valve was associated with less PPM implantation. Regarding PPM follow-up, patients who had a PPM due to advanced AVB presented a significantly higher percentage of VP than did patients receiving it for other reasons, such as LBBB plus low-grade AVB. Altogether, this report highlights the importance of further evidence to more accurately select patients with LBBB that benefit from PPM implantation and those who do not, strengthening the ambulatory close monitoring strategy to promptly recognize advanced AVB events in these patients. Furthermore, results in advanced AVB patients reinforce the rationale of implanting more physiological modes of pacing in this group.
